# Hypoxia treatment of Parkinson’s disease may disrupt the circadian system

**DOI:** 10.1186/s12883-023-03270-y

**Published:** 2023-06-19

**Authors:** Olivier Coste, Yvan Touitou

**Affiliations:** 1 Hôpital d’Instruction des Armées, Pathologie du Sommeil, Lyon, France; 2 Unité de Chronobiologie, Fondation Rothschild, 75019 Paris, France

**Keywords:** Parkinson’s disease, Hypoxia, Rhythm desynchronization, Internal clock

## Introduction

We have read the recent article by Janssen Daalen et al. titled "Multiple N-of-1trials to investigate hypoxia therapy in Parkinson’s disease: study rationale and protocol" [1] with great interest and would like to comment on it. The rationale for using hypoxia therapy in Parkinson’s disease (PD) and a detailed study protocol are presented in this paper.

The rationale of their prospective study is based on clinical observations. Sometimes, PD symptoms are alleviated by altitude exposure. The authors suggest that hypoxia could play a role and present different mechanisms with short-term and long terms effects. The short-term effects may be related mainly to an increase of dopamine release by substantia nigra mediated by an activation of HIF1 and in turn of tyrosine hydroxylase, a key-enzyme in dopamine synthesis. Sympathetic activation may play also a significant role. Specific protocols of hypoxia exposure (hypoxia conditioning) have been demonstrated to be neuroprotective. These protocols often rely on repeated short cycles of hypoxia exposure interspersed with normoxia or hyperoxia during several weeks [2, 3].

## Methodological considerations

The protocol by Janssen Daalen et al. is focused on the short-term effects of hypoxia. Twenty individuals with PD will experience five 45 min-sessions in a randomized order with different oxygenation conditions, i.e. continuous normoxia (FiO2 = 20.9%) as placebo, continuous hypoxia at 2000 m (FiO2 = 16.3%), continuous hypoxia at 4000 m (FiO2 = 12.7%), intermittent hypoxia with 5 × 5-min at 2000 m (FiO2 = 16.3%), interspersed with 5-min normoxic recovery, and intermittent hypoxia with 5 × 5-min at 4000 m (FiO2 = 12.7%), interspersed with 5-min normoxic recovery. Although the timing of hypoxic exposures was not provided, they appear to be scheduled mainly in the morning.

The duration of hypoxic exposure is quite brief in this exploratory study and seems therefore safe for study participants. This hypoxia protocol may be nevertheless suboptimal. Indeed, protocols for similar applications in neurological diseases in humans usually apply similar cycles but multiple times (often around 15 x) over several weeks [3]. Nevertheless, the authors do not take into account that hypoxia may also alter the circadian time structure, whereas there are some published data suggesting that circadian rhythm disruption, in addition to being a symptom of neurodegeneration, might also be a potential risk factor for developing PD [4].

## Discussion

Indeed, we studied the circadian alteration induced by a diurnal 8-h mild hypobaric hypoxia simulating a flight in a pressurized cabin of a civil and military aircraft in the experiments we led a few years ago on twenty healthy young males [5–10]. The following was the experimental design: The male volunteers were exposed to a cabin altitude of 8000 ft (2400 m) in a hypobaric chamber for 8 h (08:00–16:00 h) and then to 12,000 ft (3600 m) four weeks later. Core body temperature (CBT) was measured using telemetry for two consecutive 24-h cycles for each hypoxic exposure (control and hypoxic exposure). Individual CBT data were fitted with a five-order moving average before statistical analysis with mixed ANOVAs after filtering out non-physiological values. During these two 24-h cycles (control and hypoxic exposure), plasma melatonin and cortisol, among other variables, were measured every 2 h in all subjects.

Following the hypoxic exposure, we discovered a phase delay in the CBT rhythm and a decrease in the nocturnal peak of plasma melatonin during the night. These effects could explain, at least in part, alterations of recovery sleep analyzed from sleep logs leading to fatigue complaints of aircrews [5, 6]. Besides, we described changes in cortisol circadian, another circadian marker, under hypoxia but without the phase delay, which we observed on CBT in our experimental conditions [7]. Under hypoxia, the 24-h profiles of most current biochemical variables were also altered, with changes in the mean plasma levels and a trend toward a phase delay at both studied altitudes [8, 9].

A complementary study using a similar experimental protocol confirmed the effects of diurnal hypoxic exposures on sleep architecture as measured by polysomnography: we discovered a more precise increase in sleep onset latency and sleep fragmentation, as well as a reduction in the total sleep period, during the two nights following the hypoxic exposure [10]. Moreover, nocturnal acute hypoxia can have detrimental consequences on breathing and sleep quality [11]. This is why the time of the day for the hypoxia intervention should be chosen to be not close to sleeping otherwise there is a real risk of interference with sleep quality mainly due to sympathetic activation.

Our conditions of hypoxic exposure are quite different from those proposed in this study (hypobaric vs. normobaric conditions and long vs. short duration of exposure) but they had quite similar degrees of hypoxia that will be employed in the proposed study. Moreover, there is no data to our knowledge dealing with the impact of a shorter exposure (i.e. 45 min in the present study) on circadian time structure. The induction of changes in circadian rhythms following short hypoxia exposure is in fact not well known, despite molecular crosstalk between hypoxia adaptation and circadian rhythm pathways. For example, HIF1, which may play a short-term positive role for improving PD symptoms [1], also interacts with clock genes at the molecular level [12]. Moreover, a number of examples in the literature point out that the internal clock can be affected even by a weak stimulus or a stimulus of short duration e.g., light, drugs [13, 14].

Another question that arises deals with the optimal timing of a brief exposure to hypoxia. Has a morning exposure to hypoxia (vs. an evening or a nocturnal exposure) the same efficiency or presents the same level of security for the patients, using a chrono-pharmacological and-therapeutical approach [15, 16]?

## Conclusion

It appears it would be important to take into account the circadian rhythmicity in a further research program, if this original preliminary study for PD treatment by hypoxia therapy presented positive outcomes.

## Data Availability

Not applicable for this section.
