# Correction to: Hypoxia treatment of Parkinson’s disease may disrupt the circadian system

**DOI:** 10.1186/s12883-023-03325-0

**Published:** 2023-07-13

**Authors:** Olivier Coste, Yvan Touitou

**Affiliations:** 1 Hôpital d’Instruction des Armées, Pathologie du Sommeil, Lyon, France; 2 Unité de Chronobiologie, Fondation Rothschild, 75019 Paris, France

**Correction: BMC Neurol 23, 234 (2023)**

10.1186/s12883-023-03270-y

Following publication of the original article [1], the authors reported an error found in the Introduction and Methodological considerations sections. The author “Janssen Daalen et al” was erroneously referred to as ‘Daalen et al.’.

The original article [1] has been updated.
